# Molecular basis for the recognition of low-frequency polyadenylation signals by mPSF

**DOI:** 10.1093/nar/gkaf890

**Published:** 2025-09-10

**Authors:** Lin Huang, Hsu-Feng Chu, Liang Tong

**Affiliations:** Department of Biological Sciences, Columbia University, New York, NY 10027, United States; Department of Biological Sciences, Columbia University, New York, NY 10027, United States; Department of Biological Sciences, Columbia University, New York, NY 10027, United States

## Abstract

The 3′-end cleavage and polyadenylation of pre-mRNAs is dependent on a key hexanucleotide motif known as the polyadenylation signal (PAS). The PAS hexamer is recognized by the mammalian polyadenylation specificity factor (mPSF). AAUAAA is the most frequent PAS hexamer and together with AUUAAA, the second most frequent hexamer, account for ∼75% of the poly(A) signals. The remaining hexamers are at low frequency (<3%), and the molecular basis for their recognition is still not known. Here, we have determined the binding affinities for most of the PAS hexamers, showing that the *K*_d_ values are generally inversely correlated with their frequency. We also observed good cleavage activity for two low-frequency hexamers, AAGAAA and AACAAA. We have determined the cryo-electron microscopy structures of human mPSF in complex with AAUAAU and AGUAAA, at 3.1 and 2.5 Å resolution, respectively. The overall binding modes of the two low-frequency hexamers are similar to that of AAUAAA, although the U3-A6 Hoogsteen base pair is disrupted in the AAUAAU hexamer. For AGUAAA, the G2 base undergoes a large conformational change, which allows it to maintain the hydrogen-bonding interaction with CPSF30 as observed with A2 and establish a new hydrogen bond to CPSF30.

## Graphical Abstract

**Graphical Abstract:**
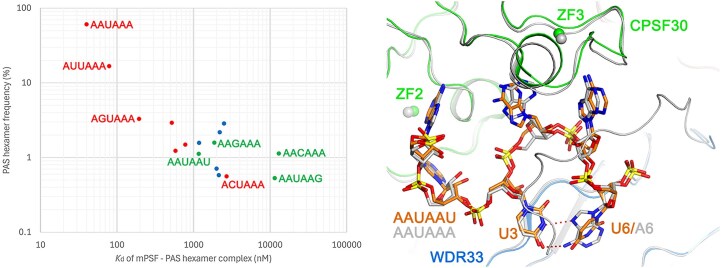

## Introduction

In eukaryotes, nascent messenger RNAs (pre-mRNAs) transcribed by RNA polymerase II (Pol II) undergo extensive processing during their maturation. The processing events include 5′ capping, splicing to remove the intronic sequences, and 3′-end cleavage and polyadenylation, with the exception of replication-dependent histone pre-mRNAs in animals, which are cleaved but not polyadenylated. The correct processing of pre-mRNAs is crucial for the production of functional mRNAs.

The specificity and efficiency of 3′-end cleavage and polyadenylation are determined by a multiprotein complex binding to defined sequence elements at the 3′ end of pre-mRNAs [1–3]. The most critical sequence element is the polyadenylation signal (PAS), a hexanucleotide typically 10–30 nucleotides (nts) upstream of the cleavage site. In mammals, AAUAAA is the most frequently seen PAS hexamer, and it is present in ∼60% of pre-mRNAs, as observed in complementary DNAs, expressed sequence tags, and 3′-end sequencing data [4–6]. The second most frequently observed PAS is AUUAAA, at ∼15% frequency. Many other PAS hexamers can also support 3′-end processing, but they are present at much lower frequency (<3%). These hexamers are often single-nucleotide variants of AAUAAA and (rarely) AUUAAA.

The PAS hexamer is recognized by the mammalian polyadenylation specificity factor (mPSF) [7, 8], which consists of four subunits: CPSF160, WDR33, CPSF30, and Fip1. Cryo-electron microscopy (cryo-EM) structures of human mPSF in complex with AAUAAA [9, 10] and AUUAAA [11] have revealed the molecular basis how these two PAS hexamers are recognized. The six nucleotides are divided into three pairs, with A1-A2 (or A1-U2) contacting the second zinc finger domain (ZF2) of CPSF30 and A4-A5 contacting the ZF3 of CPSF30. The U3-A6 bases form a Hoogsteen base pair, which is flanked by Phe43 in the N-terminal segment of WDR33 on one face and Phe153 in the WD40 domain of WDR33 on the other.

The AAUAAA PAS hexamer has high affinity for the mPSF, with dissociation constant (*K*_d_) in the low nanomolar range (∼3 nM) based on fluorescence anisotropy binding assays [12, 13]. The *K*_d_ for the AUUAAA hexamer is about six-fold higher, while a *K*_d_ for the less frequent hexamers that were examined (GAUAAA, AAUGAA, AAUACA) could only be estimated to be >500 nM due to the inability to observe a complete binding curve in the assays [13]. Our earlier attempts at determining the structure of human mPSF in complex with the GAUAAA or AAGAAA PAS hexamer were also not successful [11].

In our earlier binding assays, we used fluorescein(FAM)-labeled RNA oligos to monitor binding to mPSF [13]. We also used unlabeled RNA oligos in competition binding assays, which suggested that the FAM label may enhance the binding of the RNA to mPSF, giving apparently higher affinity for the RNA. An extreme case was the FAM-labeled oligo containing the AAGAAA hexamer, which gave a *K*_d_ of 16 nM [13]. This is supported by our failure to observe this oligo in the EM studies [11]. To obtain unbiased binding affinity measurements, we need to use unlabeled RNAs in competition fluorescence anisotropy binding assays.

The less frequent PAS hexamers also appear to have lower cleavage activity in processing assays. Especially, RNAs with AAGAAA as the poly(A) signal generally show little or no cleavage activity with nuclear extracts [14–18]. Recently, the human 3′-end cleavage and polyadenylation machinery was reconstituted using all recombinant proteins, mPSF, mCF (mammalian cleavage factor), CstF (cleavage stimulation factor), CFIIm (cleavage factor II), RBBP6, and PAP [19–21]. The cleavage activity of the AAGAAA [19] or AACAAA [20] hexamer with this reconstituted machinery is also very low.

Here, we have determined the *K*_d_ values for most of the known poly(A) signals in their binding to human mPSF. We used much higher concentrations of the RNA oligos in the competition fluorescence anisotropy assays, allowing us to observe full binding curves. We identified assay conditions that showed good cleavage activities for the AAGAAA and AACAAA poly(A) signals, consistent with their ability to support pre-mRNA 3′-end processing. We have also determined the cryo-EM structures of human mPSF in complex with the low-frequency PAS hexamers AAUAAU and AGUAAA. The overall binding modes of the two low-frequency hexamers are similar to that of AAUAAA, although the U3-A6 Hoogsteen base pair is disrupted in the AAUAAU hexamer. There are no interactions between U3 and U6, and A4-A5 also has weaker interactions with CPSF30. For AGUAAA, the G2 base undergoes a large conformational change, which allows it to maintain the hydrogen-bonding interaction with CPSF30 as observed with A2 and establish a new hydrogen bond to ZF2 of CPSF30.

## Materials and methods

### Protein expression and purification

All the recombinant proteins were expressed in baculovirus-infected Tni (*Trichoplusia ni*) insect cells (Expression Systems) as described earlier [9, 21, 22]. For human mPSF, full-length CPSF160, Fip1, and CPSF30 were inserted into pFastBac (438-A) vector (Addgene, Plasmid #55218) [23]. WDR33 (residues 1–572) was inserted into pFastBac (438-C) vector (Addgene, Plasmid #55220), which carried an amino-terminal His_6_-MBP tag followed by a TEV protease cleavage site. Fip1, WDR33, and CPSF160 were then combined into CPSF30–pFastBac (438-A) by three rounds of Gibson assembly (NEB, E2611L) [21, 24].

For human mCF, full-length CPSF100 and CPSF73 were cloned into the pSPL vector, and symplekin (residues 538–1110) was cloned into pFL vector with 6xHis tag at the N-terminus [22]. These two vectors were then fused by Cre recombinase.

For human CstF, full-length CstF77 was inserted into 438-C, and CstF64 and CstF50 were inserted into 438-A. Then, the three subunits were combined into 438-C by Gibson assembly with a His_6_-MBP tag at the N-terminus of CstF77 [21].

For human CFIIm, full-length Clp1 and Pcf11 were inserted into pFastBac (438-C) vector, with a His_6_-MBP tag at the N-terminus of Pcf11 [21].

Human RBBP6 (residues 1–399) and full-length PAP were inserted into the pFL vector with a 6xHis tag at the N-terminus, and the D115A mutant of PAP was generated by site-directed mutagenesis polymerase chain reaction [21].

For human CFIm, full-length CFIm25 and CFIm68 were inserted into the pFastBac (438-C) vector, both with a His_6_-MBP tag at the N-terminus.

All proteins were expressed and purified following protocols described earlier [21, 22]. Recombinant bacmids used for Bac-to-Bac baculovirus of all the complexes were generated in *Escherichia coli* DH10EMBacY competent cells (Geneva Biotech). Recombinant baculoviral stocks were produced in Sf9 insect cells (Expression Systems) by transfection using Cellfectin II (Thermo Fisher Scientific). P0 viruses were produced by adherent culturing the infected Sf9 insect cells at 27°C for 4 days. P1 viruses used for large-scale infection were amplified in 30 ml Sf9 insect cells at 27°C for 3 days and harvested using centrifugation at 1,100 rpm for 10 min. Two hundred fifty milliliters Tni insect cells (mid-log phase of growth corresponding to a cell density at 1.5–2 × 10^6^ viable cells/ml) were infected with a suitable amount of P1 virus (generally based on the volume ratios of 50:1–200:1) at 27°C for 2.5 days with constant shaking at 130 rpm to express the recombinant proteins. Cell pellets were harvested by centrifugation at 1,100 rpm for 10 min.

For purification, the harvested cell pellets were resuspended and lysed by sonication in 100 ml lysis buffer containing 20 mM Tris (pH 8.0), 250 mM NaCl, 5% (v/v) glycerol, 0.2% (v/v) Triton, 10 mM β-ME (β-mercaptoethanol), and one protease inhibitor cocktail tablet (Sigma), followed by centrifugation at 13,000 rpm for 35 min at 4°C to remove the cell debris. The supernatant was incubated with pre-equilibrated Ni-NTA beads (Qiagen) for 1 h at 4°C. The beads were washed with at least 50 bed volumes of wash buffer containing 20 mM Tris (pH 8.0), 250 mM NaCl, 20 mM imidazole, and 10 mM β-ME. The proteins were eluted with buffer containing 20 mM Tris (pH 8.0), 100 mM NaCl, and 250 mM imidazole, and the fractions were pooled and further purified by 1 ml HiTrap QHP anion exchange chromatography column (Cytiva) and Superose 6 10/300 gel filtration chromatography column (Cytiva) with the running buffer 20 mM Tris (pH 8.0), 150 mM NaCl, and 2 mM dithiothreitol (DTT). The peak fractions were collected and ran on 12% sodium dodecyl sulfate–polyacrylamide gel electrophoresis to check the purity. The high-quality fractions were pooled and concentrated by Millipore centrifugal filter (with a molecular-weight cutoff size of 100 kDa). The final concentration of the proteins is 5 μM. The sample was divided into 10 μl aliquots, flash-frozen in liquid nitrogen, and stored at −80°C for cryo-EM and *in vitro* assays.

### RNA oligos

The RNA oligos, possibly with FAM or TAMRA (tetramethylrhodamine) label at the 5′ or 3′ end, were purchased from Integrated DNA Technologies. The sequences of the oligos are given in Table 1 and Supplementary Table S1.

**Table 1.** Binding affinity of PAS hexamers to human mPSF

RNA sequence	Frequency (%)	*K*_d_ (nM)	Ratio
FAM-UGCAAUAAACAA	60.76	19	0.5
UGCAAUAAACAA	60.76	40	1
UGCAUUAAACAA	16.76	79	2
UGCAGUAAACAA	3.29	194	5
UGCUAUAAACAA	2.92	520	13
UGCAAUAUACAA	2.85	2,500	62
UGCAAUACACAA	2.18	2,200	55
UGCAAGAAACAA	1.58	1,870	47
UGCAAUGAACAA	1.57	1,180	30
UGCCAUAAACAA	1.49	780	20
UGCGAUAAACAA	1.23	580	14
UGCAACAAACAA	1.13	13,000	325
UGCAAUAAUCAA	1.12	1,170	29
UGCAAUAGACAA	0.71	2,000	50
UGCAUUAUACAA	0.58	2,140	54
UGCACUAAACAA	0.56	2,700	68
UGCAAUAAGCAA	0.53	11,500	288
(AUUACA)	0.40	n. d.	–
(AACAAG)	0.34	n. d.	–

The PAS hexamers are underlined. Frequency indicates the frequency the hexamer is annotated as a poly(A) signal in known human mRNAs. Ratio indicates fold change in the *K*_d_ value relative to that of the AAUAAA oligo. n. d., not done.

### Fluorescence anisotropy binding assays

Fluorescence anisotropy assays were performed using a BioTek Synergy Neo2 microplate reader. For the direct binding assay, 3 nM FAM-AAUAAA oligo (Table 1) was used as the probe, and mPSF was serial diluted down from 300 nM to 0.04 nM. For competitive binding assays, each 12-mer unlabeled PAS oligo was serial diluted down from 100 μM to 0.5 nM, the FAM-AAUAAA oligo was at a concentration of 3 nM and mPSF at 30 nM. All the mixtures were incubated on ice for 45 min in 50 μl volume containing 20 mM Tris (pH 8.0), 150 mM NaCl, 10 mM DTT, 100 nM bovine serum albumin, and 0.01% (v/v) NP-40. Forty microliters of samples were then transferred to a Corning 384-well solid black microplates (Fisher Scientific) at room temperature and used for measuring the fluorescence. All experiments were conducted in triplicates.

The direct binding data were fitted to a single-site binding equation, and competitive binding data were fitted according to [25] to determine the *K*_d_ and generate the binding curves.

### Cleavage assays

The cleavage assays followed the protocols described earlier [21]. The RNA substrates were at a concentration of 125 nM (unless noted otherwise). The protein components mPSF, mCF, CstF, CFIIm (full-length Pcf11), and PAP (D115A) were at a concentration of 125 nM, and RBBP6 (residues 1–399) was at 500 nM concentration. The 10 μl reaction mixtures were incubated at 30°C for 2–13 h in a buffer containing 20 mM HEPES (pH 7.5), 75 mM KCl, 4 mM DTT, 2.5% (w/v) PEG 6000, 2.5 mM ATP, and 1 U RNasin Plus RNase Inhibitor (unless otherwise specified). The reactions were quenched by the addition of an equal volume of RNA Loading Dye (2X) (NEB) and heated at 95°C for 10 min. The denatured samples were loaded onto a 15% (w/v) denaturing polyacrylamide gel containing 8 M urea and electrophoresed at 250 V for 50 min. The cleavage products of the FAM-labeled RNA were imaged using a ChemiDoc system (Bio-Rad) with a 488-nm excitation wavelength. For the unlabeled RNA substrates, the urea gel was stained with SYBR Gold (1×) for 30 min at room temperature and viewed with ChemiDoc system (Bio-Rad) with an appropriate setting for SYBR Gold.

### EM data collection and processing

The cryo-EM grid preparation and data collection were conducted as previously described [9, 11]. To prepare the complex sample, 0.4 mg/ml (1.4 μM) mPSF was mixed with 25 μM 12-mer RNA oligo (AAGAAA, UAUAAA, AAUGAA, AGUAAA, or AAUAAU) in a buffer containing 20 mM Tris (pH 8.0), 150 mM NaCl, and 2 mM DTT, incubated on ice for 1 h, and centrifuged at 12 000 rpm for 10 minutes, before loading onto glow-discharged Au-flat Gold 300 mesh 1.2/1.3 grids (Electron Microscopy Sciences). All cryo-EM grids were prepared with a Vitrobot Mark IV plunge freezer (Thermo Scientific) set at 22°C and 90% humidity in the Simons Electron Microscopy Center (SEMC) at the New York Structure Biology Center (NYSBC). Four microliters sample was applied to each EM grid, which was blotted for 8 s at a blot force of −2 with 2 s wait time and plunged into liquid ethane pre-cooled by liquid nitrogen.

The EM girds were screened on a Glacios microscope (Thermo Scientific) at Columbia University Cryo-Electron Microscope Center. The presence of the RNA in the complex could often be visualized in 3D reconstructions based on movies collected during screening, and those grids that did not have bound RNA were not pursued further. Datasets used for structure determination were collected on a Titan Krios electron microscope operated at 300 kV at NYSBC, equipped with a Gatan K3 Summit detector imaging system and a Gatan Quantum GIF LS energy filter.

The dataset on the AGUAAA complex was collected at the National Center for Cryo-EM Access and Training (NCCAT). The microscope was operated at 105,000× nominal magnification. The images were recorded at a pixel size of 0.8256 Å in a super-resolution mode, and a calibrated pixel size of 0.4128 Å was used for processing. Movies were collected using Leginon at a dose rate of 44.04 e^−^/Å^2^/s with a total exposure of 1.20 s, for an accumulated dose of 52.85 e^−^/Å^2^. Intermediate frames were recorded every 0.03 s for a total of 40 frames per movie. A total of 6395 movies were collected at a defocus range of −0.6 to −2.0 μm. A 30° tilt during data collection was employed to address preferred particle orientation.

The dataset on the AAUAAU complex was collected at SEMC. The microscope was operated at 81,000× nominal magnification and a pixel size of 0.856 Å. Movies were collected using Leginon [26] at a dose rate of 31.97 e^−^/Å^2^/s with a total exposure of 1.60 s, for an accumulated dose of 51.15 e^−^/Å^2^. Intermediate frames were recorded every 0.04 s for a total of 40 frames per movie. A total of 5772 movies were collected at a defocus range of −0.5 to −2.0 μm. A 30° tilt during data collection was also employed.

Cryo-EM data processing and 3D reconstruction were carried out using CryoSPARC [27]. A flow chart of the data processing is showed in Supplementary Fig. S1. The AGUAAA and AAUAAU datasets were processed in a similar manner, and the final 3D reconstruction was at 2.53 and 3.07 Å resolution for the AGUAAA and AAUAAU datasets, respectively.

### Model building and refinement

The structure of the AAUAAA complex (PDB code: 6DNH) [9] was used as the starting model and docked into the cryo-EM density map of the AGUAAA complex using UCSF ChimeraX [28]. Model was iteratively refined through manual adjustments in Coot [29] and real-space refinement in PHENIX [30]. The model quality was validated by MolProbity program [31] and wwPDB validation system. Figures were prepared using ChimeraX and PyMOL (www.pymol.org). The AAUAAU structure model was obtained similarly. The statistics of data processing and structure refinement are summarized in Table 2.

**Table 2.** Summary of cryo-EM and structure refinement statistics

Structure	Human mPSF– AAUAAU complex	Human mPSF– AGUAAA complex
Data collection and processing		
Voltage (kV)	300	300
Electron exposure (e^−^/Å^2^)	51.15	52.85
Movies	5,772	6,395
Initial particle images	2,142,227	3,406,541
Final particle images	292,202	529,257
Symmetry imposed	C1	C1
Map resolution (Å)	3.07	2.53
FSC threshold	0.143	0.143
Map sharpening *B* factor (Å^2^)	−105	−93
Structure refinement		
No. of protein residues	1,647	1,660
No. of RNA nts	6	6
No. of metal ions	3	3
rms deviations		
Bond lengths (Å)	0.002	0.003
Bond angles (°)	0.57	0.50
Clash score	7	5
Poor rotamers (%)	0.35	1.24
Ramachandran plot		
Favored (%)	93.68	95.21
Allowed (%)	6.32	4.97
Disallowed (%)	0	0
PDB entry code	9OXS	9OXE

## Results and discussion

### Binding affinity of PAS hexamers for mPSF

To find all the known PAS hexamers that are present in human pre-mRNAs, we collected the annotated poly(A) signals in all the known mRNAs in the human genome database (GRCh38 from GenBank RefSeq FTP site, downloaded on 7 January 2024). A total of 18 PAS hexamers were identified from this collection (Table 1), at frequencies similar to those reported earlier [4–6]. The 18 hexamers include AAUAAA, 14 single-nucleotide variants of AAUAAA (including AUUAAA), and 2 single-nucleotide variants of AUUAAA (AUUAUA and AUUACA). The remaining hexamer, AACAAG, has two nucleotide changes from AAUAAA and is at the lowest frequency.

For pre-mRNAs with only one annotated PAS hexamer, AAUAAA, is at a much higher frequency, 73%, probably ensuring the cleavage of the pre-mRNAs, while the other hexamers are at substantially lower frequencies (Supplementary Table S2). On the other hand, for pre-mRNAs with more than two annotated PAS hexamers, the intermediate ones have much lower frequency for AAUAAA (44.6%), consistent with the importance of the low-frequency hexamers for alternative polyadenylation. Interestingly, for pre-mRNAs with two or more annotated PAS hexamers, the first and last hexamers have similar abundance of the different hexamers.

For binding studies, we added 3 nts (UGC) at the 5′ end and 3 nts (CAA) at the 3′ end of the hexamer, thereby generating a 12-mer for each PAS. These flanking sequences are derived from the SV40 late pre-mRNA substrate (Supplementary Table S1, and see below), and they are not directly involved in binding mPSF based on the current structures [9–11]. For example, the 12-mer for AAUAAA has the sequence UGCAAUAAACAA, and we will refer to it as the AAUAAA oligo (or AAUAAA 12-mer) here for simplicity (Table 1). We also obtained a FAM-labeled AAUAAA oligo, FAM-AAUAAA, so that we could monitor binding to mPSF by fluorescence anisotropy. All the *K*_d_ values were determined through competition binding assays against this FAM-AAUAAA 12-mer, to avoid the effects of the FAM label on the binding. The two PAS hexamers with the lowest frequency, AUUACA and AACAAG, were not studied in these binding assays.

**Figure 1.**
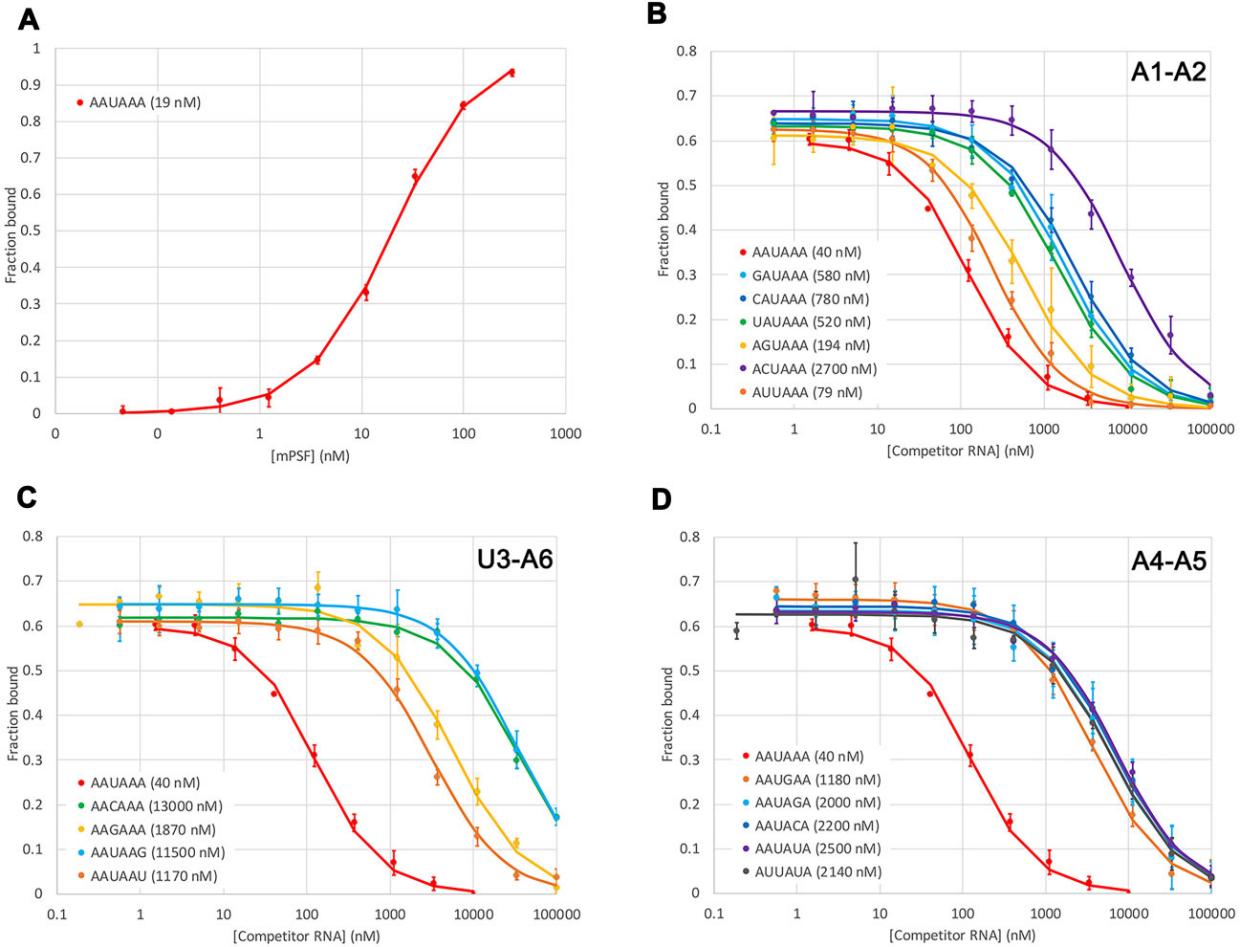
Binding affinity of human mPSF with various PAS hexamers. (**A**) Direct fluorescence anisotropy binding assay of human mPSF with FAM-AAUAAA 12-mer RNA. (**B**) Competition fluorescence anisotropy binding assays of mPSF with PAS oligos having variations at the A1-A2 positions. (**C**) Competition fluorescence anisotropy binding assays of mPSF with PAS oligos having variations at the U3-A6 Hoogsteen base pair. (**D**) Competition fluorescence anisotropy binding assays of mPSF with PAS oligos having variations at the A4-A5 positions. The *K*_d_ values of the PAS oligos are indicated in parentheses. Curves represent fitting of binding data to the theoretical equation, with error bars showing ±1 standard deviation from triplicate experiments.

We realized that the inability to observe complete binding curves in our earlier assays was likely due in part to the fact that the highest concentration of the unlabeled RNA we used in competition was 5 μM [13]. Expecting the weak affinity of these RNAs for mPSF, a much higher RNA concentration would be necessary to observe competition against the AAUAAA RNA. For the assays reported here, we used up to 100 μM unlabeled RNA and observed good binding signals (Fig. 1). We were able to fit the observed binding curves to the theoretical equation [25] and obtain the *K*_d_ value for each RNA (Table 1).

**Figure 2.**
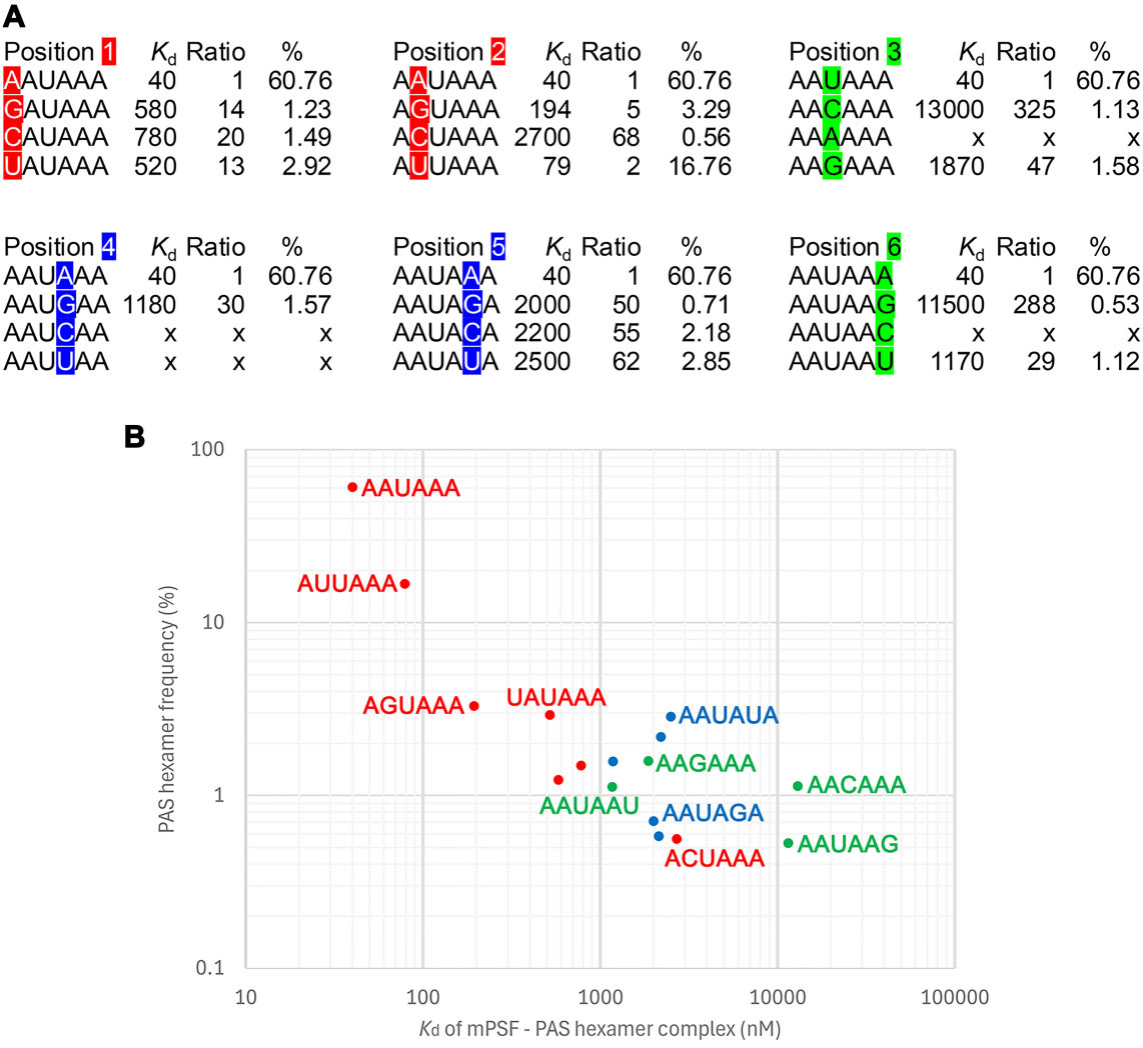
Inverse correlation between the *K*_d_ values of PAS oligos and their frequencies in human mRNAs. (**A**) The *K*_d_ values (*K*_d_, in nM) and fold change relative to AAUAAA (Ratio) for variations at each of the six PAS hexamer positions are shown, with the position having the variations highlighted in colors. Red: AAUAAA and variations at the A1-A2 positions; Blue: variations at the A4-A5 positions; Green: variations at the U3-A6 positions. %: frequency of the PAS hexamer in human mRNAs. x: a hexamer that has not been annotated as a PAS. (**B**) The distribution of the frequencies of the PAS hexamers versus their *K*_d_ values in a log–log plot. A linear inverse correlation can be seen for many of the hexamers. Selected PAS hexamers are labeled.

We first carried out direct binding assays with the FAM-AAUAAA 12-mer and determined its *K*_d_ value as 19 nM (Fig. 1A). This value is higher than that (0.3 nM) we reported earlier [13], but those assays were carried out with a different RNA as well as a different mPSF (WDR33 covering residues 1–425 rather than 1–572 here, and Fip1 covering residues 159–200 rather than full-length here). Including only the fragment of Fip1 in mPSF is known to enhance the binding affinity to RNA [12]. For the comparisons here, it is the ratio of the *K*_d_ values (Table 1 and Fig. 2A), rather than the exact *K*_d_ values themselves, that is important. Moreover, having a higher *K*_d_ for the FAM-labeled RNA was helpful for observing the complete binding curve in our competition binding assays.

We then carried out competition binding assays for the 16 unlabeled PAS oligos. The *K*_d_ value for the AAUAAA oligo was determined to be 40 nM, roughly two-fold higher than the FAM-AAUAAA 12-mer. As the six PAS nucleotides are divided into three groups based on the binding mode, the binding data are presented here in these three groups as well. For single-nucleotide variants at the A1-A2 positions, the *K*_d_ values range between 79 nM for AUUAAA (2-fold higher than AAUAAA) and 2,700 nM for ACUAAA (67-fold higher than AAUAAA) (Figs 1B and 2A). At the U3-A6 positions, the *K*_d_ values range between 1,170 nM for AAUAAU (29-fold higher than AAUAAA) and 13,000 nM for AACAAA (320-fold higher than AAUAAA) (Figs 1C and 2A). At the A4-A5 positions, the *K*_d_ values range between 1,180 nM for AAUGAA (30-fold higher than AAUAAA) and 2,500 nM for AAUAUA (62-fold higher than AAUAAA) (Figs 1D and 2A).

The observed *K*_d_ values of the various PAS oligos are generally inversely correlated with their frequency (Fig. 2B), suggesting that one of the reasons for the different frequencies of the PAS hexamers is their affinity for mPSF. In fact, the inverse correlation appears to be rather linear in the log–log plot, with a few outliers for hexamers at very low frequency.

The binding curves suggest that the affinity of the PAS hexamers for mPSF is less sensitive to single-nucleotide variations at the A1-A2 positions (Fig. 1B), with many variants having *K*_d_ values <15-fold of that for AAUAAA (Fig. 2A and B). In contrast, the affinity is quite sensitive to variations at the A4-A5 positions (Fig. 1D), with most variants having *K*_d_ values >50-fold higher than that for AAUAAA (Fig. 2A). Two of the single-nucleotide variants at the A4 position (AAUCAA and AAUUAA) have not been annotated as poly(A) signals, suggesting that it is important to have a purine base at the A4 position. The U3-A6 positions are also very sensitive to variations (Fig. 1C), and the two largest losses in affinity (∼300-fold) are observed here (Fig. 2A and B). All the variations here are expected to abolish the U3-A6 Hoogsteen base pair, but the AAGAAA oligo has a 47-fold loss in affinity, as compared to 325-fold for AACAAA. Two of the single-nucleotide variants at the U3-A6 positions (AAAAAA and AAUAAC) have not been annotated as poly(A) signals in human mRNAs.

### Cleavage assays with RNAs containing AAGAAA and AACAAA PAS hexamers

**Figure 3.**
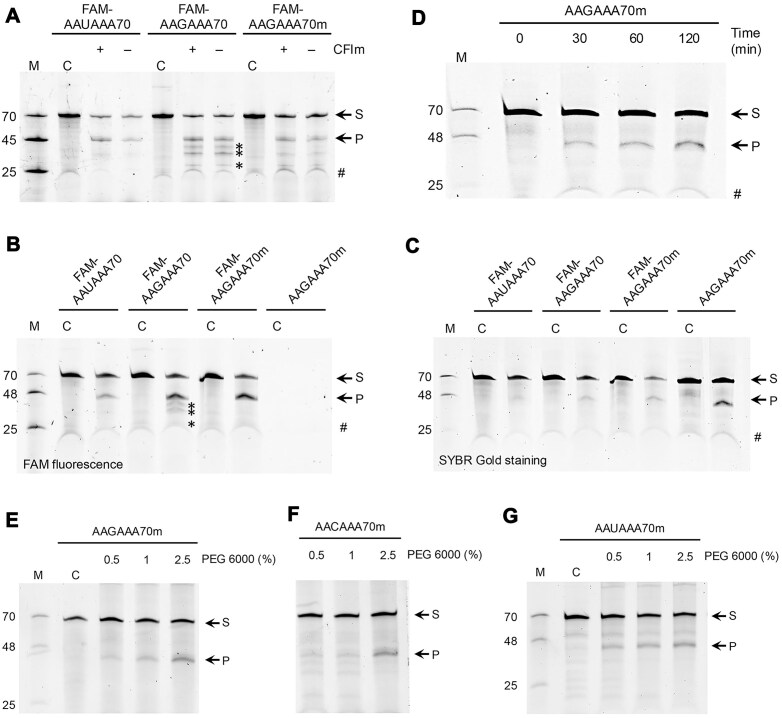
Cleavage assays with RNA substrates containing AAUAAA, AAGAAA, or AACAAA poly(A) signal. (**A**) Cleavage assays with the indicated 70-mer RNA substrates. Some of the reactions also contained CFIm, as indicated. The asterisks indicate smaller products with the FAM-AAGAAA70 substrate. The reaction mixtures were incubated for 13 h. M: marker containing FAM-labeled 70-, 45-, and 25-mers; C: control, which has RNA only, no proteins; S: the 70-mer RNA substrate; P: the 45-mer cleavage product; #: an artificial feature in the gel. (**B**) Gel image visualized with FAM fluorescence of cleavage reactions with the indicated RNA substrates. The reaction mixtures were incubated for 2 h. The AAGAAA70m lanes are empty as there is no FAM label on this RNA. M: marker containing FAM-labeled 70-, 48-, and 25-mers. (**C**) The same gel as panel (B), visualized after SYBR Gold staining. The unlabeled 45-mer 5′ product runs slightly faster than the FAM-labeled 45-mer product due to the lack of the FAM label. (**D**) Time course of the cleavage reaction with the AAGAAA70m RNA substrate. Cleavage assays with the AAGAAA70m (**E**), AACAAA70m (**F**), or AAUAAA70m (**G**) RNA substrate in the presence of increasing concentrations of PEG 6000. Most of the experiments in this figure have been carried out at least three times, with similar results. The reactions were at 30°C temperature and contained 125 nM RNA and 125 nM protein factors (except RBBP6, which was at 500 nM). Assays in panels (A)–(D) contained 2.5% (w/v) PEG 6000.

We carried out cleavage assays with a 70-mer model substrate labeled with FAM at the 5′ end based on the SV40 late pre-mRNA (to be referred to as FAM-AAUAAA70 here). This RNA contains AAUAAA as the poly(A) signal, a downstream element, and a UGUA motif upstream of the AAUAAA (Supplementary Table S1). The cleavage reaction generates a FAM-labeled 45 nts 5′ product and an unlabeled 25 nts 3′ product. We observed good cleavage activity with this substrate (Fig. 3A), similar to what we reported earlier [21]. To test the activity of the AAGAAA PAS hexamer in this assay, we obtained another FAM-labeled RNA with AAUAAA changed to AAGAAA (FAM-AAGAAA70; Supplementary Table S1). Surprisingly, we also observed good cleavage activity with this RNA substrate (Fig. 3A). Noting that the weaker interaction with mPSF for AAGAAA may make the binding at the UGUA motif more important, we also included CFIm in some of the reactions. The presence of CFIm did not make a noticeable difference to the cleavage activity with the FAM-AAGAAA70 or the FAM-AAUAAA70 substrate (Fig. 3A).

Another unexpected observation with the FAM-AAGAAA70 substrate is that the reaction generated lower levels of several smaller 5′ FAM-labeled products compared to the FAM-AAUAAA70 RNA (Fig. 3A). To understand how these smaller products could have been generated, we examined the sequence of the RNA carefully and realized that the substrate contained a second, low-frequency PAS hexamer, AUUAUA (Table 1), between the UGUA motif and the AAUAAA PAS hexamer (Supplementary Table S1). We reasoned that this second PAS hexamer may have mediated the cleavage to generate the smaller products. To test this hypothesis, we obtained another RNA oligo, this one with AUUAUA mutated to ACCACA (FAM-AAGAAA70m; Supplementary Table S1). The amounts of smaller products were greatly reduced in cleavage assays with this new substrate (Fig. 3A), confirming that AUUAUA was serving as a poly(A) signal in addition to AAGAAA in this substrate. With AUUAUA as the PAS, cleavage could occur at a few sites between AAGAAA and the original cleavage site, after CA or UA dinucleotide (Supplementary Table S1), generating the smaller products.

The good activity of the FAM-AAGAAA70 substrate was surprising, given the low activity of this PAS hexamer in cleavage assays reported earlier. Noting that the FAM label enhances the binding of the RNA to mPSF, we tested the activity of an RNA substrate without FAM label but with the same sequence (AAGAAA70m; Supplementary Table S1) and found cleavage activity even without the FAM label (Fig. 3B and C). We carried out a time course experiment with this substrate and observed steady increase in the product over time (Fig. 3D).

We tested an RNA substrate with AACAAA as the PAS hexamer (AACAAA70m; Supplementary Table S1) and also found good cleavage activity (Supplementary Fig. S2), even though this PAS hexamer has 325-fold higher *K*_d_ for mPSF compared to AAUAAA (Fig. 2A).

We discovered that the PEG 6000 component in the reaction buffer was important for the activities with the AAGAAA and AACAAA hexamers. At 2.5% (w/v) PEG 6000, good activities were observed with AAGAAA (Fig. 3A) and AACAAA (Fig. 3F), while at 1% or 0.5% PEG 6000, the activities were much weaker. In comparison, the activity of the AAUAAA hexamer was much less sensitive to the PEG 6000 concentration in the assay buffer (Fig. 3G). As a crowding agent, PEG 6000 may facilitate and/or stabilize the interaction between the RNA substrate and the machinery, thereby enhancing the activity toward the AAGAAA and AACAAA substrates, which have weaker interactions with mPSF. To test this hypothesis, we carried out fluorescence anisotropy binding assays in the presence of PEG 6000 but did not see a significant enhancement in affinity (Supplementary Fig. S2).

The observation of cleavage activity with the AAGAAA and AACAAA hexamers is consistent with them being able to serve as poly(A) signals. Differences in assay conditions may explain why much lower activity was observed earlier with these two hexamers. The assays with AAGAAA included a crowding agent, trimethylamine oxide, but they contained 3 nM mPSF, 25 nM mCF, and CstF [19]. In comparison, our assays contained 125 nM mPSF, mCF, CstF, and the RNA substrate, and the higher concentrations should favor the reaction. The assays with AACAAA used 50 nM CPSF and 100 nM CstF, but a crowding agent was not present [20]. The cleavage activity of the low-frequency AAGAAA and AACAAA hexamers is highly dependent on the PEG 6000 concentration while that of AAUAAA is not, which may be a consequence of their different affinity for mPSF. Further studies will be needed to fully characterize how these low-frequency hexamers mediate the cleavage of the pre-mRNA substrate. The recognition of the downstream element in the pre-mRNA by CstF is also a crucial component to the cleavage reaction.

### Structure of human mPSF in complex with the AAUAAU poly(A) signal

We next attempted to determine the structures of mPSF in complex with some of the low-frequency PAS hexamers. Noting their weak affinity, we included higher concentrations of the RNA in the EM sample (up to 40 μM, with mPSF at 1.4 μM). However, we were unable to observe RNA density with the AAGAAA (1.58% frequency, 1,870 nM *K*_d_), UAUAAA (2.92%, 520 nM), and AAUGAA (1.57%, 1,180 nM) 12-mers. On the other hand, we were able to determine the structure of mPSF in complex with the AAUAAU (1.12%, 1,170 nM) or AGUAAA (3.29%, 194 nM) 12-mer. The AGUAAA hexamer is the third most frequent PAS (and has the third lowest *K*_d_), while the AAUAAU hexamer has much lower frequency (Table 1). It is not clear why we were able to observe the binding of this hexamer but not the AAGAAA, UAUAAA, and AAUGAA hexamers. Especially, UAUAAA is the fourth most frequent hexamer.

**Figure 4.**
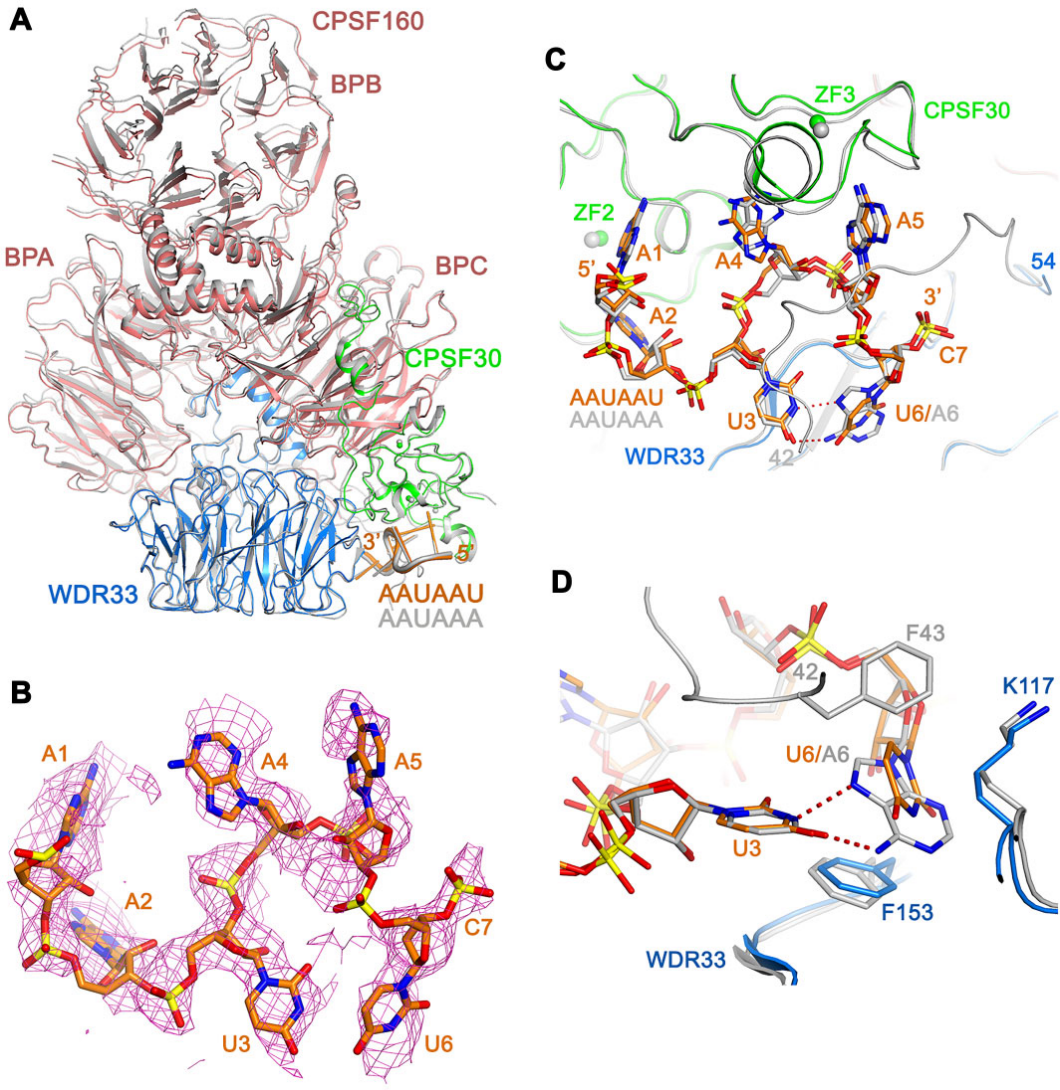
Recognition of the AAUAAU hexamer by human mPSF. (**A**) Overlay of the structure of the AAUAAU quaternary complex (CPSF160: deep salmon, WDR33: marine, CPSF30: green, RNA: orange) with that of the AAUAAA complex (gray). The superposition is based on WDR33. (**B**) EM density for the AAUAAU hexamer. (**C**) Overlay of the overall binding modes of AAUAAU (in color) and AAUAAA (gray). (**D**) Overlay of the binding mode of U3-U6 in the AAUAAU complex (in color) with that of U3-A6 in the AAUAAA complex (gray).

The structure of human mPSF in complex with the AAUAAU12 oligo has been determined at 3.1 Å resolution by cryo-EM (Fig. 4A, Supplementary Figs S1 and S3, and Table 2). The atomic model has good agreement with the EM density and the expected bond lengths, bond angles, and other geometric parameters. The overall structure of the mPSF–AAUAAU complex is similar to that of the mPSF–AAUAAA complex (Fig. 4A) [9], with root mean square (rms) distance of 0.76 Å for 1,644 equivalent Cα atoms in CPSF160, WDR33, and CPSF30. For the structure comparisons reported here, an overlay based on WDR33 is used, which gives a better superposition of the AAUAAU and AAUAAA molecules due to the proximity of WDR33 to the RNA. With this superposition, a change in the position of the CPSF160 β-propeller B (BPB) is noticeable between the two complexes (Fig. 4A). A change in the position of BPB was also observed in the AUUAAA complex [11], and in fact the AAUAAU complex overall structure is much more similar to that of the AUUAAA complex (Supplementary Fig. S3).

The AAUAAU hexamer plus the phosphate of the following nucleotide are observed in the EM density (Fig. 4B). The A1, A2, and U3 nucleotides have good density, while A4, A5, and U6 have weaker density, especially the A5 base and the backbone phosphate of U6. The overall binding mode of AAUAAU is similar to that of AAUAAA, and the overall conformations of WDR33 and CPSF30 in the binding site are similar as well (Fig. 4C).

As expected, the replacement of A6 with U6 in the AAUAAU hexamer disrupted the U3-A6 Hoogsteen base pair (Fig. 4C). The binding mode of U3 is nearly the same in the two structures (Fig. 4D). The U6 base rotates by ∼30° relative to A6 and is not hydrogen-bonded with the U3 base. In addition, Phe43 in the N-terminal extension of WDR33, which stacks against the A6 base in the AAUAAA complex, is disordered in the AAUAAU complex. In fact, good quality EM density only begins to be observed starting at residue 54 of WDR33 (Fig. 4C), and Lys46, Arg47, and Arg49 are also disordered. These positively charged side chains have interactions with other backbone phosphates in the AAUAAA complex.

The A4-A5 dinucleotide has weaker interactions with ZF3 of CPSF30 in the AAUAAU complex (Supplementary Fig. S3). The A4 base can no longer hydrogen bond to ZF3 due to a change in its conformation. The A5 base has very weak EM density, and Phe98 of CPSF30 that stacks with this base also has weak density. The EM density for the entire ZF3 is also weaker, suggesting that both ZF3 and the A4-A5 dinucleotide are more flexible in the AAUAAU complex. In comparison, the A1-A2 dinucleotides and ZF2 of CPSF30 have stronger EM density and most of the interactions in this region are similar in the two complexes (Supplementary Fig. S3).

Overall, the structure of the AAUAAU complex confirms the disruption of the U3-A6 Hoogsteen base pair. As a result, there are no interactions between U3 and U6, and the A4-A5 dinucleotides have weaker interactions with CPSF30 ZF3. In addition, a segment of the N-terminal extension of WDR33 is disordered. This segment is also disordered in the structure of CPSF160–WDR33 binary complex [9]. These structural disturbances are consistent with the weaker affinity of this hexamer for mPSF.

### Structure of human mPSF in complex with the AGUAAA poly(A) signal

**Figure 5.**
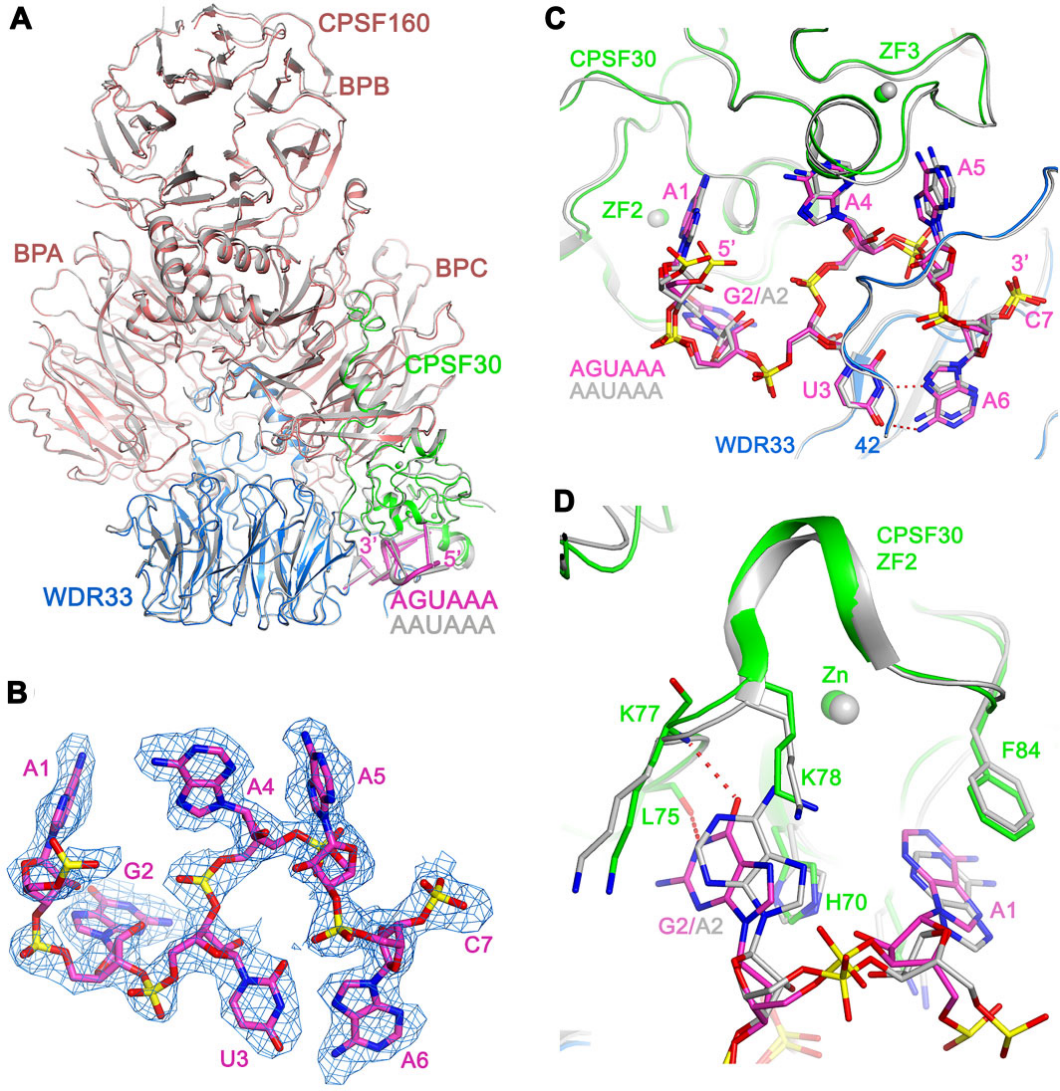
Recognition of the AGUAAA hexamer by human mPSF. (**A**) Overlay of the structure of the AGUAAA quaternary complex (CPSF160: deep salmon, WDR33: marine, CPSF30: green, RNA: magenta) with that of the AAUAAA complex (gray). The superposition is based on WDR33. (**B**) EM density for the AGUAAA hexamer. (**C**) Overlay of the overall binding modes of AGUAAA (in color) and AAUAAA (gray). (**D**) Overlay of the binding mode of A1-G2 in the AGUAAA complex (in color) with that of A1-A2 in the AAUAAA complex (gray).

We have determined the structure of human mPSF in complex with the AGUAAA PAS hexamer at 2.5 Å resolution (Fig. 5A, Supplementary Figs S1 and S4, and Table 2). The overall structure of the mPSF–AGUAAA complex is similar to that of the mPSF–AAUAAA complex [9], with rms distance of 0.58 Å for 1,656 equivalent Cα atoms in CPSF160, WDR33, and CPSF30 (Fig. 5A). We will also use an overlay based on WDR33 in the descriptions below. The position of the CPSF160 BPB is similar to that in the AAUAAA complex.

Good quality EM density was observed for the AGUAAA hexamer and the phosphate of the following nucleotide (Fig. 5B). The overall binding mode of the AGUAAA PAS is similar to that of AAUAAA, and the overall conformations of WDR33 and CPSF30 in the binding site are similar as well (Fig. 5C).

A large conformational change is observed for the G2 base compared to A2 (Fig. 5D). The N1 atom of A2 in the AAUAAA complex is a hydrogen-bond acceptor with the backbone amide of Lys77 of CPSF30 ZF2. In comparison, the N1 of G2 is a hydrogen-bond donor, and the conformational change brings the O6 atom of G2 into a similar position as the N1 of A2, thereby restoring this hydrogen bond. This also allows the N1 of G2 to make a hydrogen bond with the carbonyl oxygen of Leu75, and this interaction is not present with A2. A rearrangement in the position of A1 base is also observed, although the interactions with ZF2 are maintained. In comparison to A1-G2, the binding modes of A4-A5 and U3-A6 are similar to those in the AAUAAA complex (Supplementary Fig. S4).

Our studies have determined the binding affinities of most of the known PAS hexamers for mPSF, revealing that the *K*_d_ values are generally inversely correlated with the frequencies of the hexamers. Overall, mPSF recognizes the two most frequent PAS hexamers, AAUAAA and AUUAAA, with high affinity, while the rare PAS hexamers exhibit 5- to 300-fold lower affinity. Variations at the A1-A2 positions are more tolerated than those at the A4-A5 positions. The interactions between A1-A2 and the ZF2 of CPSF30 may have more plasticity, allowing for the variations, which is observed in our earlier structure of the AUUAAA complex [11] and that of the AGUAAA complex here.

We have observed for the first time good cleavage activity for the AAGAAA and AACAAA hexamers, consistent with them being able to support 3′-end processing. The differences in the assay conditions are likely the reason why these hexamers were found to have very low activity earlier. In addition, AAGAAA is the poly(A) signal for an immuoglobulin locus in murine cells [32], and AACAAA can mediate the cleavage of a model pre-mRNA substrate in human cells [33], indicating that they do function as PAS hexamers *in vivo*.

Remarkably, our studies show that the SV40 late pre-mRNA substrate actually has two PAS hexamers in it, AAUAAA and the low-frequency AUUAUA. When the AAUAAA is changed to AAGAAA, both PAS hexamers appear to be able to direct cleavage of the RNA substrate, with multiple products generated. On the other hand, with AAUAAA in the RNA, it dominates the cleavage reaction and only a single product is generated.

We have also determined the first structures of human mPSF in complex with low-frequency PAS hexamers, AAUAAU and AGUAAA. The structure of the AAUAAU complex indicates that the U3-A6 Hoogsteen base pair in the AAUAAA complex is not essential for binding to mPSF, which may have implications for the binding of the AAGAAA and AACAAA hexamers. The overall binding modes of these low-frequency hexamers are similar to those of AAUAAA and AUUAAA. Our attempts at determining the structures of three other low-frequency PAS hexamers were not successful. It may be necessary to develop new protocols to reveal the binding modes of the other low-frequency hexamers.

## Supplementary Material

gkaf890_Supplemental_File

## Data Availability

The atomic coordinates and the EM maps have been deposited at the Protein Data Bank and EMDB, with accession codes 9OXE/EMD-70974 (AGUAAA complex) and 9OXS/EMD-70993 (AAUAAU complex).
