# Supplementary material for: Molecular basis for the recognition of low-frequency polyadenylation signals by mPSF
Source: Nucleic Acids Res. 2025 Sep 10;53(17):gkaf890. doi: 10.1093/nar/gkaf890 (PMC12421377; doi:10.1093/nar/gkaf890)
Supplement: gkaf890_Supplemental_File [file gkaf890_supplemental_file.pdf]

Table S1

Sequences of RNA substrates used in this study

RNA	Sequence
FAM-AAUAAA70	FAM-CUUUAUUUGU AACCAUUAUA AGCUGCAAUA AACAAGUUAA CAACA↓ACAAU UGCAUUCAUU UUAUGUUUCA
FAM-AAGAAA70	FAM-CUUUAUUUGU AACCAUUAUA AGCUGCAAGA AACAAGUUAA CAACA↓ACAAU UGCAUUCAUU UUAUGUUUCA
FAM-AAGAAA70m	FAM-CUUUAUUUGU AACCACCACA AGCUGCAAGA AACAAGUUAA CAACA↓ACAAU UGCAUUCAUU UUAUGUUUCA
AAGAAA70m	CUUUAUUUGU AACCACCACA AGCUGCAAGA AACAAGUUAA CAACA↓ACAAU UGCAUUCAUU UUAUGUUUCA
AACAAA70m	CUUUAUUUGU AACCACCACA AGCUGCAACA AACAAGUUAA CAACA↓ACAAU UGCAUUCAUU UUAUGUUUCA
AAUAAA70m	CUUUAUUUGU AACCACCACA AGCUGCAAUA AACAAGUUAA CAACA↓ACAAU UGCAUUCAUU UUAUGUUUCA

The downward arrow indicates the site of cleavage. The PAS hexamer is colored red, downstream sequence element in blue, and upstream UGUA motif in green. A second PAS hexamer AUUAUA is colored orange, which is mutated to ACCACA in some of the RNAs. Possible cleavage sites from the AUUAUA hexamer in FAM-AAGAAA70 are indicated in orange.

Table S2

PAS hexamer frequencies (%)

PAS sequence	Overall[a]	Single[a]	First[a]	Intermediate[a]	Last[a]
AAUAAA	60.76	72.99	58.19	44.56	58.24
AUUAAA	16.76	14.39	18.40	19.27	16.54
AGUAAA	3.29	2.40	3.09	5.08	3.40
UAUAAA	2.92	1.57	3.09	4.95	3.14
AAUAUA	2.85	1.36	2.83	4.73	3.55
AAUACA	2.18	1.04	2.25	3.53	2.69
AAGAAA	1.58	0.89	2.20	2.06	1.55
AAUGAA	1.57	0.83	1.45	2.52	2.01
CAUAAA	1.49	0.98	1.34	2.40	1.68
GAUAAA	1.23	0.66	1.17	2.44	1.20
AACAAA	1.13	0.76	1.55	1.50	0.95
AAUAAU	1.12	0.51	1.08	1.79	1.52
AAUAGA	0.71	0.28	0.74	1.36	0.82
AUUAUA	0.58	0.21	0.60	1.22	0.62
ACUAAA	0.56	0.38	0.60	0.75	0.63
AAUAAG	0.53	0.27	0.47	0.93	0.68
AUUACA	0.40	0.20	0.55	0.64	0.35
AACAAG	0.34	0.28	0.38	0.28	0.43

[a]The ‘Overall’ frequency is for all the annotated hexamers. The ‘Single’ frequency is for the hexamer in pre-mRNAs with only one annotated poly(A) signal. The ‘First’ frequency is for the first hexamer in pre-mRNAs with more than one annotated poly(A) signals. The ‘Intermediate’ frequency is for the intermediate hexamers in pre-mRNAs with more than two annotated poly(A) signals, excluding the first and last hexamers. The ‘Last’ frequency is for the last hexamer in pre-mRNAs with more than one annotated poly(A) signals.

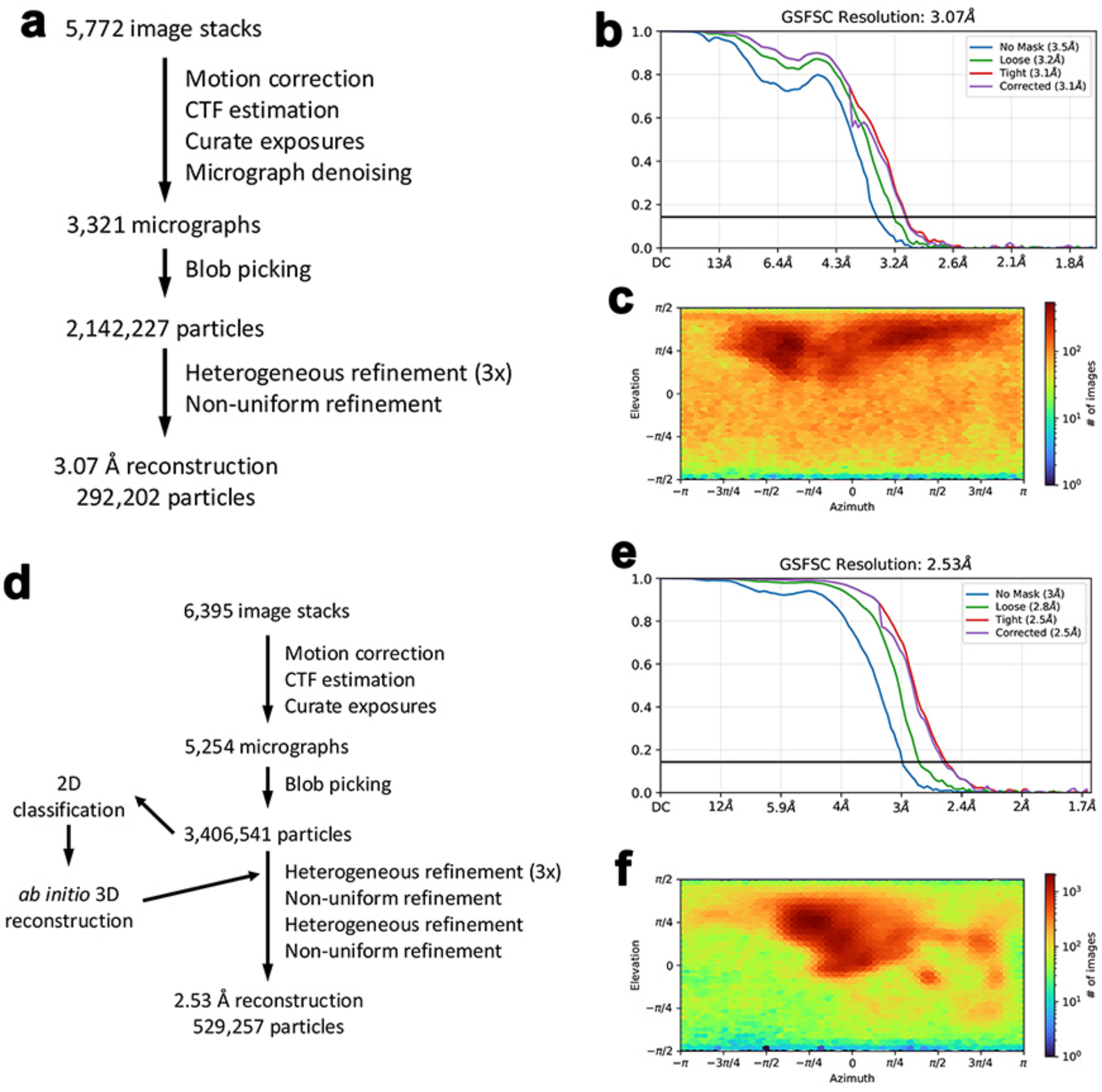

Fig. S1. Single-particle cryo-EM analysis of human mPSF in complex with RNA. **(a).** Flow chart of the cryo-EM data processing for the mPSF-AAUAAU complex. The initial volumes for heterogeneous refinement came from those for the AGUAAA complex. **(b).** Fourier shell correlation curves for the final reconstruction. **(c).** Orientations of particles used in the refinement for the final cryo-EM reconstruction. **(d).** Flow chart of the cryo-EM data processing for the mPSF-AGUAAA complex. **(e).** Fourier shell correlation curves for the final reconstruction. **(f).** Orientations of particles used in the refinement for the final cryo-EM reconstruction.

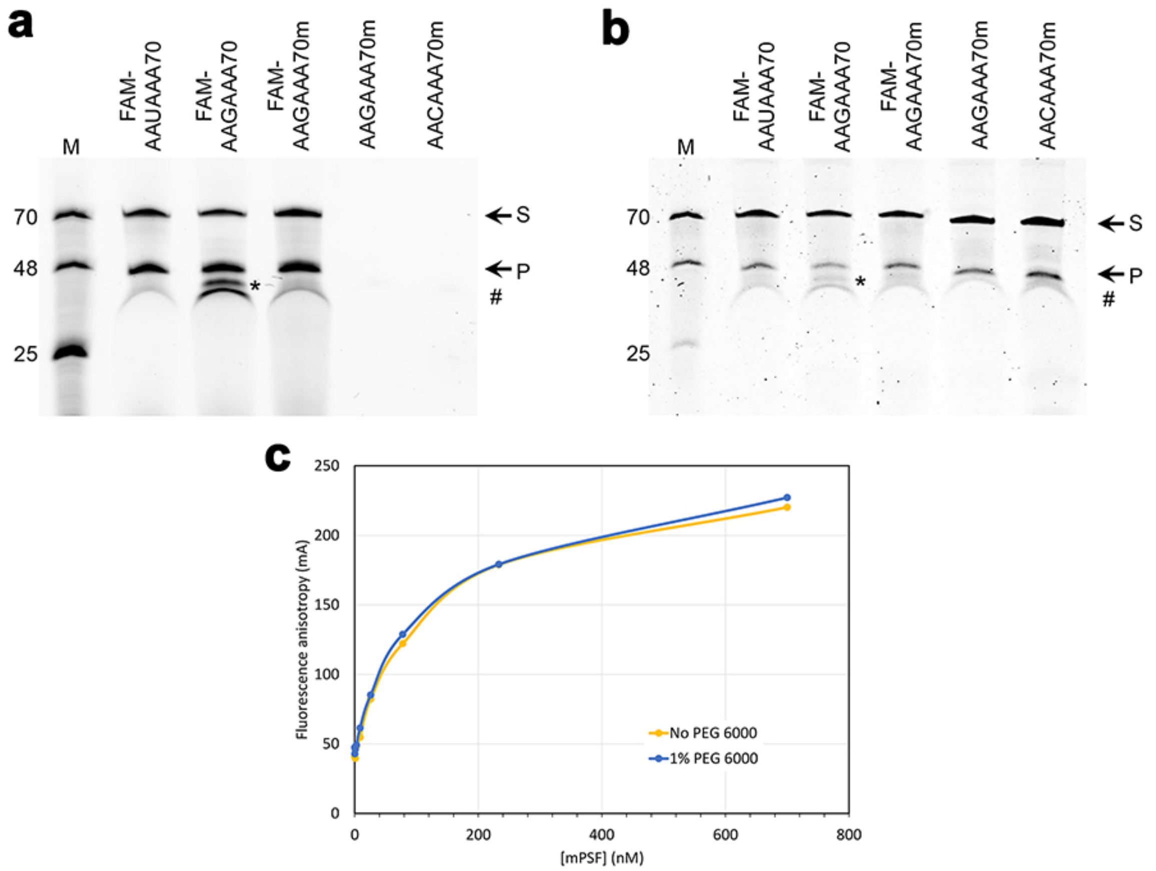

Fig. S2. Cleavage assays with RNA substrates containing AAUAAA, AAGAAA and AACAAA poly(A) signals. **(a).** Gel image visualized with FAM fluorescence of cleavage reactions with the indicated RNA substrates. **(b).** The same gel visualized after SYBR Gold staining. The unlabeled 45-mer 5′ product (P) runs slightly faster than the FAM-labeled 45-mer product. **(c).** Fluorescence anisotropy binding assays with FAM-labeled 70-mer SV40 late pre-mRNA substrate in the absence or presence of 1% PEG 6000. A binding assay with 2.5% PEG 6000 could not be performed as 700 nM mPSF precipitated at that PEG concentration.

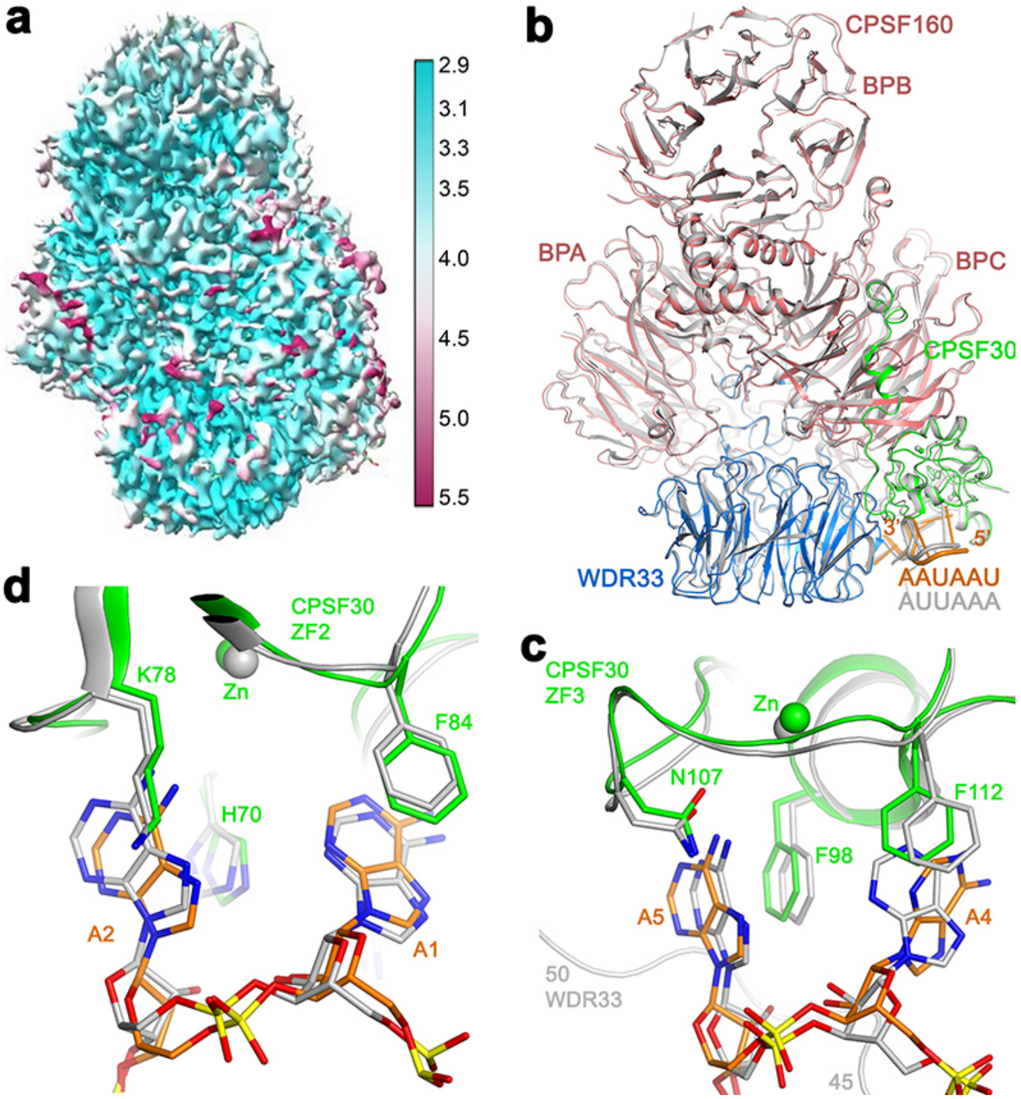

Fig. S3. (a). Local resolution map for human mPSF in complex with AAUAAU RNA. **(b).** Overlay of the structure of the AAUAAU complex (in color) with that of the AUUAAA complex (gray). **(c).** Overlay of the binding mode of A4-A5 in the AAUAAU complex (in color) with that in the AAUAAA complex (gray). **(d).** Overlay of the binding mode of A1-A2 in the AAUAAU complex (in color) with that in the AAUAAA complex (gray).

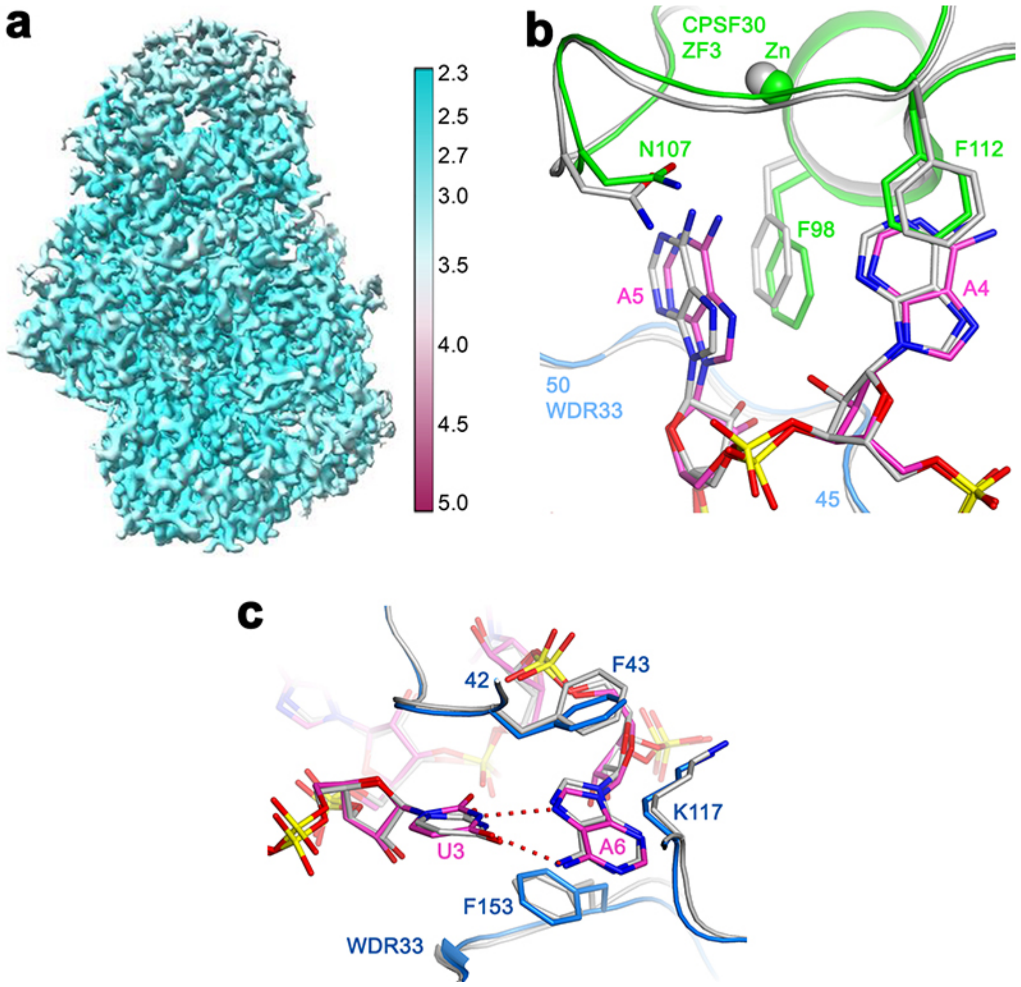

Fig. S4. (a). Local resolution map for human mPSF in complex with AGUAAA RNA. **(b).** Overlay of the binding mode of A4-A5 in the AGUAAA complex (in color) with that in the AAUAAA complex (gray). **(c).** Overlay of the binding mode of U3-A6 in the AGUAAA complex (in color) with that in the AAUAAA complex (gray).
